# External validity of docetaxel triplet trials in advanced gastric cancer: are there patients who still benefit?

**DOI:** 10.1007/s10120-020-01116-x

**Published:** 2020-09-24

**Authors:** Paula Jimenez-Fonseca, Alberto Carmona-Bayonas, Eva Martínez de Castro, Ana Custodio, Carles Pericay Pijaume, Raquel Hernandez, Gema Aguado, Natalia Castro Unanua, Juana María Cano, Flora López, Marcelo Garrido, Ana Fernández Montes, Laura Visa, Manuel Sánchez Cánovas, María Luisa Limón, Nieves Martínez Lago, Paola Pimentel, Alicia Hurtado, Aitor Azkárate, Federico Longo, Marc Diez, Aranzazu Arias-Martinez, Tamara Sauri, Alfonso Martín Carnicero, Monserrat Mangas, Marta Martín Richard, Mónica Granja, Avinash Ramchandani, Carolina Hernández Pérez, Paula Cerdá, Aitziber Gil-Negrete, Mariona Calvo, Rosario Vidal Tocino, Javier Gallego

**Affiliations:** 1 Medical Oncology Department, Hospital Universitario Central de Asturias, ISPA, Avenida Roma s/n, Oviedo, Spain; 2 Hematology and Medical Oncology Department, Hospital Universitario Morales Meseguer, University of Murcia, IMIB, CP13/00126, PI17/0050 (ISCIII& FEDER), Murcia, Spain; 3 Fundación Séneca (04515/GERM/06), Murcia, Spain; 4 Medical Oncology Department, Hospital Universitario Marqués de Valdecilla, IDIVAL, Santander, Spain; 5 Medical Oncology Department, Hospital Universitario La Paz, CIBERONC CB16/12/00398, Madrid, Spain; 6 Medical Oncology Department, Hospital Universitario Parc Tauli, Sabadell, Spain; 7 Medical Oncology Department, Hospital Universitario de Canarias, Tenerife, Spain; 8 Medical Oncology Department, Hospital Universitario Gregorio Marañón, Madrid, Spain; 9 Medical Oncology Department, Complejo Hospitalario de Navarra, Pamplona, Spain; 10 Medical Oncology Department, Hospital General Universitario de Ciudad Real, Ciudad Real, Spain; 11 Medical Oncology Department, Hospital Universitario Doce de Octubre, Madrid, Spain; 12 Medical Oncology Department, Pontificia Universidad Católica de Chile, Santiago de Chile, Chile; 13 Medical Oncology Department, Complejo Hospitalario de Orense, Orense, Spain; 14 Medical Oncology Department, Hospital Universitario El Mar, Barcelona, Spain; 15 Hematology and Medical Oncology Department, Hospital Universitario Morales Meseguer, Murcia, Spain; 16 Medical Oncology Department, Hospital Universitario Virgen del Rocío, Seville, Spain; 17 Medical Oncology Department, Complejo Hospitalario Universitario de A Coruña, Coruña, Spain; 18 Medical Oncology Department, Hospital Santa Lucía, Cartagena, Spain; 19 Medical Oncology Department, Hospital Universitario Fundación Alcorcón, Madrid, Spain; 20 Medical Oncology Department, Hospital Universitario Son Espases, Mallorca, Spain; 21 Medical Oncology Department, Hospital Universitario Ramón and Cajal, Madrid, Spain; 22 Medical Oncology Department, Hospital Universitario Vall d’Hebron, Barcelona, Spain; 23 Pharmacy Department, Hospital Universitario Central de Asturias, Oviedo, Spain; 24 Medical Oncology Department, Hospital Clinic, Barcelona, Spain; 25 Medical Oncology Department, Hospital San Pedro, Logroño, Spain; 26 Medical Oncology Department, Hospital Galdakao-Usansolo, Galdakao-Usansolo, Spain; 27 Medical Oncology Department, Hospital Universitario Santa Creu i Sant Pau, Barcelona, Spain; 28 Medical Oncology Department, Hospital Universitario Clínico San Carlos, Madrid, Spain; 29 Medical Oncology Department, Hospital Universitario Insular de Gran Canaria, Las Palmas de Gran Canaria, Spain; 30 Medical Oncology Department, Hospital Universitario Nuestra Señora de la Candelaria, Tenerife, Spain; 31 Medical Oncology Department, Centro Médico Tecknon, Barcelona, Spain; 32 Medical Oncology Department, Hospital Universitario de Donostia, San Sebastián, Spain; 33 Medical Oncology Department, Catalan Institute of Oncology, L’Hospitalet, Spain; 34 Medical Oncology Department, Complejo Asistencial Universitario de Salamanca, IBSAL, Salamanca, Spain; 35 Medical Oncology Department, Hospital General Universitario de Elche, Elche, Spain

**Keywords:** Bayesian model, Chemotherapy, Gastric cancer, Docetaxel, Survival

## Abstract

**Background:**

The purpose of our study was to develop an online calculator to estimate the effect of docetaxel triplets (DPF) in first line of advanced gastric cancer (AGC), and to assess the external validity of docetaxel trials in individual patients.

**Methods:**

The study includes patients with HER2(-) AGC treated with platin and fluoropyrimidine (PF) or with DPF in first line. Treatment effect and interactions were assessed using Bayesian accelerated failure time models.

**Result:**

The series comprises 1376 patients; 238 treated with DPF and 1138 with PF between 2008 and 2019. DPF was associated with increased progression-free survival (PFS) and overall survival (OS) with time ratio (TR) 1.27 (95% credible interval [CrI], 1.15–1.40), and TR 1.19 (95% CrI, 1.09–1.27), respectively. Serious adverse events were more common with DPF, particularly hematological effects (32% vs 22%). Younger participants received greater DPF dose density without achieving greater disease control, while severe toxicity was likewise higher. DPF yielded superior OS in Lauren intestinal (TR 1.27, 95% CrI, 1.08–1.11) vs diffuse subtype (TR 1.17, 95% CrI, 1.09–1.24) and the probability of increasing OS > 15% was 90% vs 67% in each subtype, respectively. The effect dwindles over time, which can be attributed to pathological changes and clinical practice changes.

**Conclusion:**

Our study confirms the effect of DPF is highly dependent on several clinical–pathological variables, with discreet and gradually declining benefit over platinum doublets in later years, at the expense of increased toxicity. These results may help to underpin the idea that external validity of AGC trials should be revised regularly.

**Electronic supplementary material:**

The online version of this article (10.1007/s10120-020-01116-x) contains supplementary material, which is available to authorized users.

## Introduction

External validity or the inferential generalizability of randomized clinical trials (RCTs) refers to the appropriateness of extrapolating trial outcomes from one population to another. It is a complex concept whose determinants are based more on clinical rather than statistical expertise [1] and on factors such as selection criteria, pathological traits, or access to new therapies [2]. Therefore, in light of the ever-changing condition of clinical practice and target populations, this inferential generalizability of past RCTs should be revisited every so often [3–5]. The apparent dwindling effect of docetaxel-containing triple-agent regimens (DPF) in the first-line treatment of advanced gastric cancer (AGC) may be illustrative of this situation.

AGC continues to be a common neoplasm with high mortality, for which palliative chemotherapy is recommended [6]. In 2006, the international TAX325 phase III RCT demonstrated the benefit of docetaxel combined with platin-fluoropyrimidine (DPF) in first line [7]. Nonetheless, the effect on overall survival (OS) of DPF over platin-fluoropyrimidine was modest (median 9.2 vs 8.6 months, hazard ratio (HR) 0.77, *P* value = 0.02) and was attained at the cost of substantially increased severe toxicity (69% vs 59%), respectively. This discreet benefit profile has diminished in successive RCTs [8, 9]. In this regard, the 2017 update of Wagner’s meta-analysis, with eight comparative studies, lowered the prospect of benefit (HR 0.86, 95% confidence interval (CI) 0.78–0.95) [9]. More recently, adding docetaxel to cisplatin and S-1, appraised in the Japanese JCOG1013 phase III RCT, did not enhance OS (HR 0.99, 95% CI, 0.85–1.16), although it did increase adverse events (e.g., grade 3–4 neutropenia, 59% vs 32%) [8].

This series of results revealing progressively smaller magnitude poses the clinician with a twofold question. The first is whether the effect of docetaxel varies on the basis of time factors, geographical or epidemiological variables. In the interim between these studies, changes in clinical practice have been reported that may have modified the effect. Chief among these changes is the introduction of trastuzumab in tumors that amplify or overexpress *human epidermal growth factor receptor*-*2* (HER2) in 2010 [10]. Given that these neoplasms are no longer treated with docetaxel-based triplet, candidates for DPF include ever since an abundance of diffuse histological subtype—precisely the ones that are most chemo-refractory [10–12]. In addition to this, in 2014, the RAINBOW RCT confirmed the benefit of paclitaxel and ramucirumab in second line, which could dilute the advantage of the docetaxel-based triplet in first line [13]. Thus, clinical guidelines now tend to recommend the dual-agent platin and fluoropyrimidine regimen for HER2-negative AGC, with DPF as an option in fit individuals who require tumor response [14, 15].

The second question is whether there are still any patients who could benefit from the DPF strategy in first line. This is germane, given the paucity of data regarding effect-modifying factors based on key variables such as histopathologic subtype or age [6]. Our study seeks to shed light on both questions. We have, therefore, attempted to reproduce the trends observed in RCTs in a national registry of AGC. Moreover, we have constructed an online calculator that depicts how individual characteristics or clinical–pathological variables have modified the effect that the addition of docetaxel has had on survival endpoints.

## Method

### Patients and study design

The participants are from the AGAMENON registry in which 34 Spanish and one Chilean center participate and that recruit consecutive cases of locally advanced, unresectable or metastatic adenocarcinoma of the stomach, gastroesophageal junction, or distal esophagus [12, 16–24].

Eligibility criteria for this analysis include being > 18 years of age, cancer that does not overexpress HER2, and first-line treatment with at least one cycle of platin and 5FU (PF) with or without docetaxel [14]. Patients who had completed a prior neoadjuvant or adjuvant treatment in the previous 6 months and those who had received taxanes as part of perioperative schedules were excluded.

The data are managed through a website (http://www.agamenonstudy.com/) that consists of filters and a system of queries to guarantee data reliability and control for missing and inconsistent data, with telephone and online monitoring (PJF).

The study was approved by a multicenter Research Ethics Committee of all the Autonomous Communities and participating hospitals and was classified as a prospective, postmarketing surveillance study by the Spanish Agency of Medicines and Medical Devices (AEMPS), and was not otherwise involved in it. All participants still alive at the time of data collection provided written, signed, informed consent.

### Variables

The main endpoint, OS, was defined as the time between treatment initiation and demise from any cause, censoring patients lost to follow-up. Progression-free survival (PFS) was defined as the interval between start of treatment and progression, as per the RECIST 1.1 criteria, death, or last follow-up. Relative dose intensity (RDI) was expressed in percentages and defined as the dose intensity (the amount of drug per unit of time, expressed as mg/m2 weekly) administered with respect to the planned amount for each schedule. To limit confounding bias, nine clinical, pathological, and laboratory confounding factors were selected, on the basis of theoretical criteria and the group’s experience from previous studies [25]. These covariates were performance status (ECOG-PS), Lauren histological classification subtype, histological grade, ascites, stage (III unresectable vs IV), liver tumor burden, type of platin (cisplatin vs oxaliplatin), neutrophil/lymphocyte ratio, age, and year of treatment [26].

To limit the confounding bias, two measures of tumor load have been contemplated: the number of organs involved and liver tumor burden. Patients’ baseline computerized tomographies were re-evaluated by the clinicians and liver tumor burden was categorized as: 0 metastasis, < 25%, 25–50%, 51–75%, and > 75% of liver volume affected by tumor tissue. The number of organs involved was defined as the number of organs (e.g., liver, lung, skeleton, lymph nodes, unresected gastric primary, etc.) affected by the disease, regardless of the number of metastases contained in each organ. Lymph metastases in distant basins were considered different organs.

### Statistics

Therapeutic effect was evaluated via a Bayesian parametric accelerated failure time (AFT) model with lognormal distribution; this model assumes that the effect of the covariates is to accelerate or decelerate the course of illness, making them suitable when the assumption of proportional hazards is not met [23]. Its coefficients have an intuitive, direct interpretation in its exponentiated form, as time ratios (TR). Thus, a time ratio equal to 2 for a binary predictor means that the median of time-to-event is doubled in the presence of this variable. With this model, the interaction between DPF, age, histopathologic subtype, and year of treatment was examined. This approach also enables historical external data to be incorporated as priors [27–30]. The prior for the therapeutic effect was based on Wagner’s meta-analysis, for OS ~ N(0.15, 0.045) and for PFS ~ N(0.27, 0.07) [9]. The interactions were appraised skeptically, e.g., ~ N(0, 0.05) for the histological subtype, to discourage subgroup effects deemed extreme [7]. An online calculator was built to obtain the model’s predictions. The trace and density plots for Markov chain Monte Carlo (MCMC) samples denoted adequate convergence (Gelman–Rubin Rhat measure < 1.1 for all the parameters). A frequentist AFT model for OS is also shown in Annex Figure 1 as comparison.

Continuous variables were assessed by restricted cubic splines. Covariates with > 25% missing data were discarded and multiple imputation was applied (fully conditional specification, on 20 imputed datasets) in the rest. The probability of dichotomous outcomes was appraised by logistic regression. All analyses were performed with the R v3.1.6 software package, with the mice, splines, and brms libraries [31–34].

## Results

### Patients

**Fig. 1:**
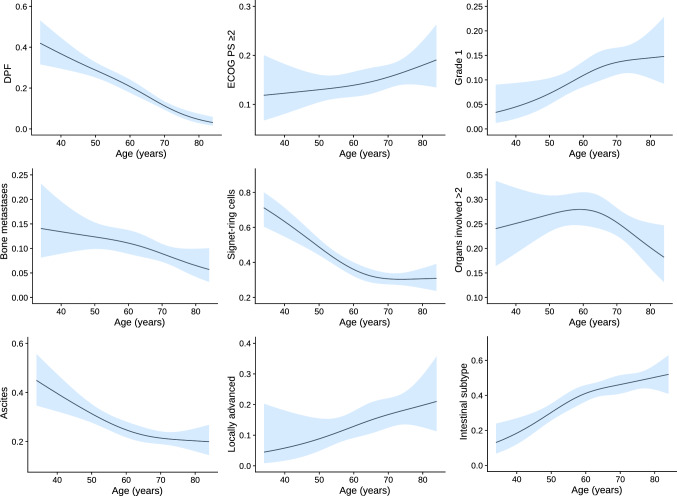
Probability of clinical–pathological variables and treatment pattern based on age. DPF, docetaxel, platinum, fluoropyrimidine; ECOG-PS, Eastern Cooperative Group Performance Status. NOTE: The estimations are derived from logistic regressions that evaluate the non-linear effect of age (restricted cubic splines with 3 knots) on binary variables

The series comprises 1376 patients; 238 treated with DPF and 1138 with PF between 2008 and 2019. Annex Table 1 details the chemotherapy schedules used. Baseline characteristics are displayed in Table 1. The most salient differences include the fact that patients treated with PF (vs DPF) tend to be older (median 66 vs 58 years), with worse functional status (ECOG-PS ≥ 2, 17% vs 7%), predominance of intestinal subtype (34% vs 26%), and less of a propensity toward peritoneal disease (45% vs 53%). Figure 1 illustrates how younger individuals are more likely to receive DPF and have tumors with distinct clinical–pathological traits (higher percentage of diffuse tumors, signet ring cells, ascites, and greater tumor burden).

**Table 1:** Baseline characteristics of patients

Baseline characteristics	Total, *n* = 1376	PF, *n* = 1138	DPF, *n* = 238
Age, median (range)	65 (20–89)	66 (20–89)	58 (22–88)
Sex, female	450 (33%)	367 (32%)	83 (35%)
ECOG-PS			
0	282 (20%)	217 (19%)	65 (27%)
1	889 (65%)	732 (64%)	157 (66%)
≥ 2	205 (15%)	189 (17%)	16 (7%)
Primary tumor site			
Esophagus	114 (9%)	102 (9%)	12 (5%)
GEJ	158 (11%)	132 (12%)	26 (11%)
Stomach	1104 (80%)	904 (79%)	200 (84%)
Histological grade			
1	120 (9%)	110 (10%)	10 (4%)
2	328 (25%)	337 (29%)	44 (18%)
3	563 (42%)	450 (40%)	113 (48%)
Not available	312 (24%)	241 (21%)	71 (30%)
Lauren classification			
Diffuse	641 (47%)	526 (46%)	115 (49%)
Intestinal	444 (32%)	383 (34%)	61 (26%)
Not available	291 (21%)	229 (20%)	62 (25%)
Signet ring cells	433 (31%)	362 (32%)	71 (30%)
Tumor stage at diagnosis, locally advanced unresectable	59 (4%)	41 (4%)	18 (7%)
Metastases sites			
Ascites	344 (25%)	288 (25%)	56 (24%)
Peritoneal	634 (46%)	508 (45%)	126 (53%)
Bone	137 (10%)	112 (10%)	25 (11%)
Lung	167 (12%)	158 (14%)	9 (4%)
Liver	486 (35%)	430 (38%)	56 (24%)
Burden of liver disease > 50%	238 (17%)	207 (18%)	31 (13%)
Number of metastatic sites > 2	349 (25%)	291 (26%)	58 (24%)
Platin			
Oxaliplatin	894 (65%)	818 (72%)	76 (32%)
Cisplatin	482 (35%)	320 (28%)	162 (68%)
Primary tumor resection	441 (32%)	360 (32%)	81 (34%)
CEA, ng/ml			
< 5	653 (47%)	521 (46%)	132 (55%)
5–10	131 (10%)	111 (10%)	20 (8%)
10–30	121 (9%)	102 (9%)	29 (12%)
> 30	232 (17%)	197 (17%)	35 (16%)
No available	239 (17%)	207 (18%)	22 (9%)
Albumin, g/dl			
> 35 g/dl	921 (67%)	757 (67%)	164 (69%)
30–35	218 (16%)	187 (16%)	31 (13%)
< 30	114 (8%)	99 (9%)	15 (6%)
No available	123 (9%)	95 (8%)	28 (12%)

DPF, docetaxel, platinum, fluoropyrimidine; ECOG-PS, Eastern Cooperative Group Performance Status; GEJ, gastroesophageal junction; PF, platinum, fluoropyrimidine

At the time of analysis, with a median follow-up of 35.3 months, 1208 progression events and 1128 deaths had been reported. Median PFS and OS for the entire sample were 5.8 (95% CI, 5.5–6.1) and 10.2 (95% CI, 9.6–10.8) months, respectively.

### Evaluation of the main effect, dose intensity, and safety profile

Median PFS was 6.5 (95% CI, 5.6–7.3) vs 5.7 months (5.4–6.12) and median OS was 11 (95% CI, 9.8–10.4) vs 10.1 months (9.4–10.7) for DPF vs PF, respectively. In the multivariable model, DPF was associated with increased PFS with TR 1.27 (95% credible interval [CrI], 1.15–1.40) and also improved OS with TR 1.19 (95% CrI, 1.09–1.27). In a sensitivity analysis, the results of the Bayesian model are robust, regardless of geographical location.

The analysis of administered doses indicates that incorporating docetaxel was at the expense of a discreet decrease in RDI for platins and fluoropyrimidines, although the mean dose intensity remained above 80% in most cases (Annex Table 2). This occurred more intensely in the elderly. Thus, in DCX/DCF (docetaxel, cisplatin, capecitabine/5FU) schemes, cisplatin RDI was 81% vs 72%, and docetaxel RDI was 81% vs 72% in individuals <75 vs> 75 years, respectively. The use of cisplatin-based regimens is less common with age; the turning point occurs around the age of 65 (Annex Figure 2).

**Fig. 2:**
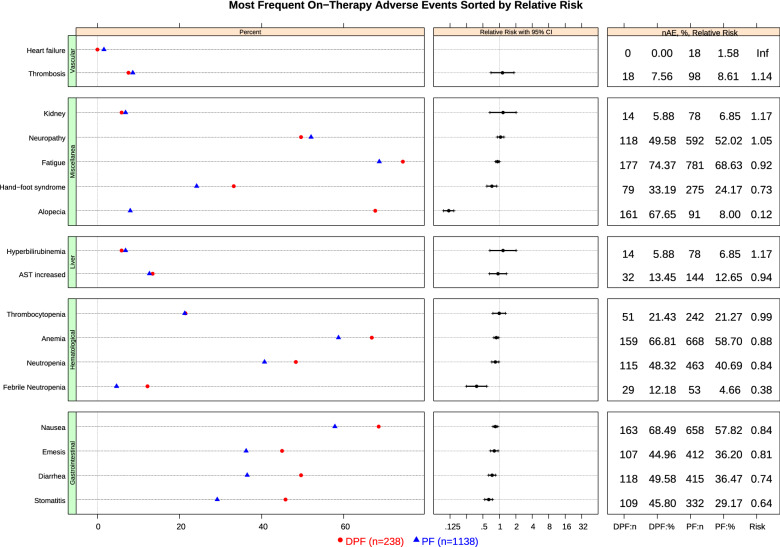
Most Frequent On − Therapy Adverse Events Sorted by Relative Risk. AE, adverse event; AST, Aspartate Aminotransferase; CI, confidence interval; DPF, docetaxel, platinum, fluoropyrimidine; n, number; PF, platinum, fluoropyrimidine

**Fig. 3:**
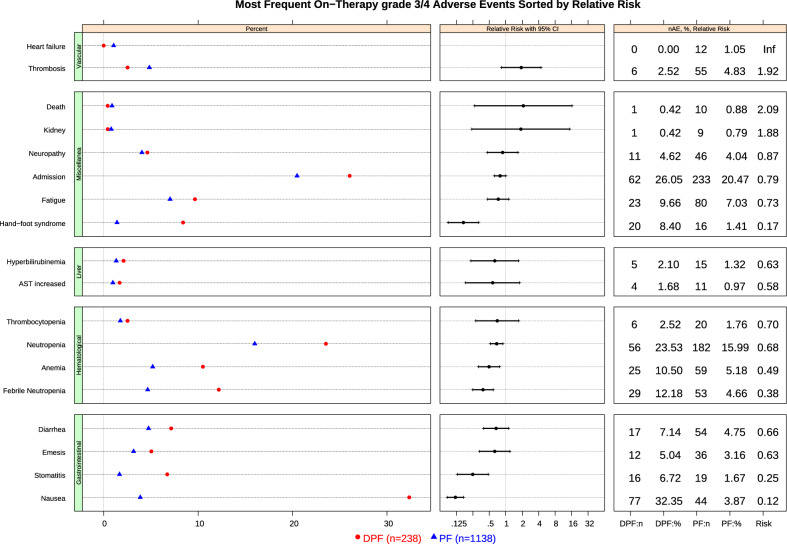
Most Frequent On − Therapy grade 3/4 Adverse Events Sorted by Relative Risk. AE, adverse event; AST, Aspartate Aminotransferase; CI, confidence interval; DPF, docetaxel, platinum, fluoropyrimidine; n, number; PF, platinum, fluoropyrimidine

Insofar as safety is concerned, DPF increased serious adverse events compared to PF, particularly hematological events (32% vs 22%), although it was also associated with more diarrhea, stomatitis, alopecia, or asthenia (Figs. 2 & 3). The attempt to intensify therapy in younger subjects yielded the highest rates of severe toxicity with DPF across the board. Accordingly, the probability of grade 3/4 toxicity for DPF was 40%, 37%, and 34%, for people aged 40, 60, and 80 years. By contrast, for PF, it was 31%, 31%, and 35%, respectively. There were fewer PF dose reductions, with 83% vs 79% oxaliplatin RDI in participants < vs ≥ 75 years, respectively.

### Conditional effects based on individual traits

We then fitted an AFT model to estimate the probability of the effect of DPF depending on individual traits. An online calculator has been designed to obtain these estimations gradually (see the online calculator: https://www.prognostictools.es/AgamenonTriplet/inicio.aspx). Annex Figure 3 shows three examples of the use of this calculator under Wagner’s meta-analysis prior.

**Fig. 4:**
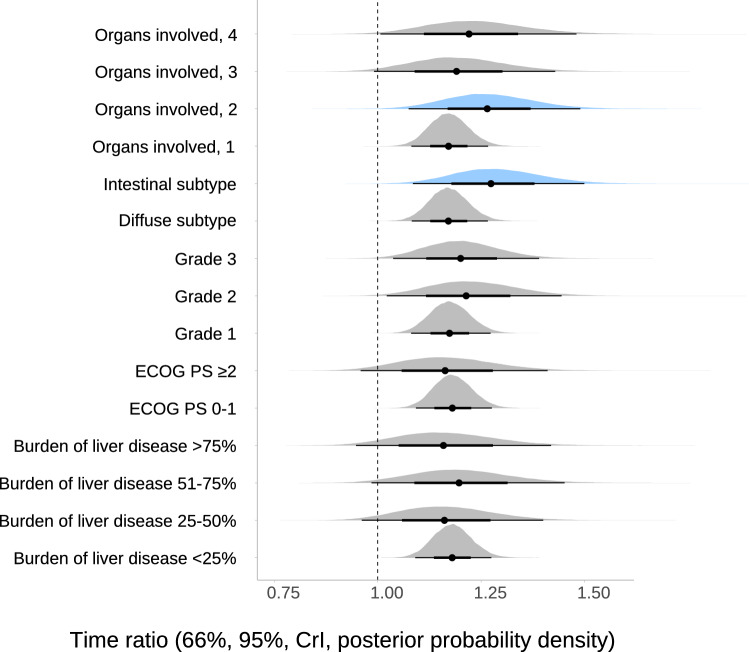
Posterior probabilities for the therapeutic effect of DPF vs PF on overall survival depending on clinical–pathological variables. CrI, credible interval. We applied specific priors for the main effect ~ N(0.15, 0.045), and skeptical priors for interactions (see methods). The density plots must be interpreted as posterior probability of the effect under each prior. Thus, the more to the right the area, the greater the benefit and the narrower it becomes, the greater the accuracy of the estimate. The results are derived from multivariable AFT models with therapeutic effect by covariate interactions. Color coding on the panels highlights the subgroups with greater effect (blue). A time ratio of more than 1 for the covariate implies that this slows or prolongs the time to event, whereas a time ratio of less than 1 indicates that an event is more likely to occur earlier. Thus, a time ratio equal to 2 would mean that the median of time to event is doubled in patients treated with DPF

The model suggests a subgroup effect based on histopathological subtype. Thus, the posterior probability of benefit differs depending on histology. Nevertheless, a discreet benefit from DPF in any of the subtypes cannot be ruled out under this supposition (Fig. 4 and Table 2).

**Table 2:** Evaluation of potentially age-dependent effect-modifying factors for OS

Variables	Specific informative prior		
	Time ratio (95%, CrI)	Posterior probability of effect size > 15% (TR > 1.15)	Posterior probability of effect size > 30% (TR > 1.30)
ECOG-PS ≥ 2	1.16 (0.99–1.36)	54%	1%
ECOG-PS 0-1	1.18 (1.10–1.25)	75%	13%
Grade 3	1.19 (1.06–1.36)	72%	14%
Grade 2	1.20 (1.05–1.40)	73%	22%
Grade 1	1.17 (1.09–1.25)	69%	1%
Intestinal subtype	1.27 (1.08–1.11)	90%	40%
Diffuse subtype	1.17 (1.09–1.24)	67%	0
Organs involved, 4	1.22 (1.04–1.43)	73%	26%
Organs involved, 3	1.18 (1.02–1.39)	65%	17%
Organs involved, 2	1.27 (1.10–1.44)	88%	37%
Organs involved, 1	1.17 (1.09–1.24)	67%	0
Burden of liver disease > 75%	1.16 (0.98–1.37)	53%	13%
Burden of liver disease 51–75%	1.19 (1.02–1.40)	66%	20%
Burden of liver disease 25–50%	1.16 (0.99–1.36)	54%	12%
Burden of liver disease < 25%	1.18 (1.10–1.25)	74%	1%

CrI, Bayesian credible interval; ECOG-PS, Eastern Cooperative Group performance status; ROPE, region of practical equivalence (effect = 0 ± 10%); TR, time ratio; OS = overall survival. The time ratios were derived from bayesian AFT lognormal models with treatment-by-covariate interactions; these models are multivariable (14 confounding factors, see Methods). The specific informative prior encompasses using the adapted evidence from Wagner’s 2017 meta-analysis (normal prior with mean = 0.15, standard deviation = 0.045 for therapeutic effect) with moderately skeptical priors for interactions (see “Methods”)

A time ratio of more than 1 for the covariate implies that this slows or prolongs the time to event, whereas a time ratio of less than 1 indicates that an event is more likely to occur earlier. Thus, a time ratio equal to 2 would mean that the median of time to event is doubled in patients treated with DPF

The posterior probability of effect sizes > 15% or 30% (TR > 1.15 or 1.30) denotes the actual probability of achieving a benefit of that magnitude (15 or 30%) or greater

**Fig. 5:**
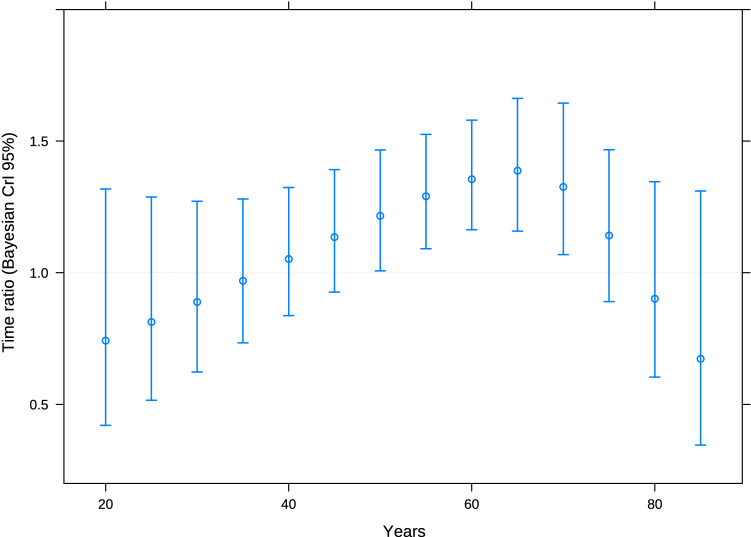
Therapeutic effect on the basis of age. CrI, credible interval. Note: Results are derived from a multivariable AFT Bayesian model, with effect by age interaction

Insofar as age is concerned, middle-aged individuals (range 50–70 years) were those who benefitted from DPF (Fig. 5). Regardless of treatment, the elderly had greater probabilities of tumor control with any treatment at 3 months, but benefited less from DPF in terms of PFS. Nor is PFS increased in the younger range of ages with docetaxel-containing triplets, given that they have more chemo-refractory tumors (Fig. 1).

Frequentist and Bayesian models based on weakly informative priors are shown in Annex Figure 1 and Annex Table 3.

### Analysis of timing on therapeutic effect

The magnitude of effect of DPF on OS has fallen over the years. In 2013, TR was 1.21 (95% CrI, 1.09–1.33) as opposed to 1.13 (95% CrI, 0.97–1.32) in 2019 (neutral prior). PFS analyses revealed similar trends. Over time, DPF is a less popular option in this series, particularly for the treatment of intestinal subtype tumors (Annex Figure 4). Since 2014, this registry is gradually collecting more treatments with paclitaxel, with or without ramucirumab, as second-line therapy (23% vs 8% after and before 2014, respectively, Annex Figure 5).

## Discussion

We have updated in a national AGC registry the external validity of the international bibliography regarding first-line therapies containing docetaxel [7–9]. Overall, we have observed that the use of triplets with docetaxel increased PFS by 27% and OS by 19% in patients with AGC. This improvement in survival endpoints is consistent with those of the data from the TAX325 trial and Wagner’s meta-analysis [7, 9], although it has come about at the expense of increased severe toxicity.

The AGAMENON registry reproduces the temporal trend seen in successive RCTs with DPF in first line [8, 9], making it a worthwhile instrument to explore the reasons for this weakening of effect and ascertain those patient groups for whom DPF combinations are still effective. Our results appear to confirm a reduction of therapeutic effect associated with DPF from 2015 onward, and contributes explanatory information regarding the fluctuation of those effects over time [7, 8]. While our data indicate that the most plausible explanation is the introduction of taxanes in second line, other aspects must be born in mind, such as the increased popularity of the oxaliplatin-containing doublet, which is observed in the literature [35, 36]. Moreover, in recent years, DPF is administered more in diffuse, treatment-resistant tumors, which may be due in part to the use of PF and trastuzumab in HER2 + cancers (most of which are intestinal subtype). In fact, the Japanese JCOG1013 RCT recruited patients with HER2-negative AGC, which possibly increased the rate of diffuse tumors, whereas theTAX325 RCT did not factor in HER2 status [7, 8].

We have elaborated an online calculator to offer an intuitive depiction of the baseline prognosis and the effect of docetaxel-containing triple-agent regimens depending on time variation and clinical–pathological factors. Due to the existence of heterogeneity of effects, the calculator aims to estimate the most likely effect of DPF as a function of covariates. The model we have adjusted here points toward the clinical profile with greater possibilities of gaining from DPF being fit subjects, in particular, aged 50–70, with intestinal tumors, and intermediate tumor burden, as the online calculator illustrates. However, the data from our registry show that younger individuals failed to attain better outcomes with DPF, as can be seen in the calculator, but did suffer a higher rate of serious adverse events. The reason can be found in the higher prevalence of aggressive, chemo-refractory tumor phenotypes in this age range (e.g., diffuse tumors, signet ring cells, peritoneal carcinomatosis, etc.). In these cases, DPF did not seemingly help to prevent progression and the result in terms of survival endpoints was more modest than in subjects aged 50–70 years who benefitted the most with this regimen [26]. Indeed, the various histopathological subtypes of gastric cancer respond differently to chemotherapy [11, 12]. Thus, the intestinal subtype, more sensitive to DPF, is more frequent in elderly individuals, paradoxically the group that tolerates this therapy worse when administered at standard recommended doses. Conversely, diffuse tumors are typical of young patients [11, 26]. Interestingly, most AGC RCTs have not stratified for histology, nor have they appraised interactions between therapeutic effect and different clinical–pathological variables [7]. For this reason, the online calculator is useful for the clinician, and adds evidence to what we already know about these combinations.

In light of this information, the AGAMENON registry points to the value of regularly re-examining and updating the external validity of RCTs, as well as to conduct geographical validations of the outcomes, particularly when the target population changes over time [10–12] or efficacious off-trial therapies entail rethinking treatment strategy [13, 37].

Among elderly subjects in the AGAMENON registry, DPF was seen to be administered with dose reductions and modifications. This mitigated the risk of toxicity in the elderly and revealed that most of the adaptations took place at the beginning, in line with the published clinical trials that have sought to modify the triplet to enhance its tolerability [38–41]. A single-phase II randomized trial (NCT00737373) has compared a docetaxel-containing triplet (FLOT) with 5FU and oxaliplatin (FLO) in an elderly population. It detected significantly increased toxicity and worse quality of life, without evidence of a gain in OS in this context [42]. On the whole, these data led to the assumption that the reduction of benefit in older ages was related to tolerance [14, 43]. In our study, modifications in elderly patients were associated with diminished therapeutic effect, similar to the AIO group’s trial, casting doubt on the usefulness of this strategy.

Our study has certain limitations inherent in a registry study, including missing values for histological subtype in 21% of the cases. The procedures of multiple imputation decrease bias, but in this case, they are associated with more conservative estimations. Second, one of the criticisms of Bayesian models is the subjectivism in selecting priors. However, the use of a perspective based on objective external data lessens this problem, and allows gradual, pragmatic answers to be attained. In this regard, Bayesian analyses have added advantages over the classical frequentist models [28], including greater accuracy of estimations and less bias. Based on the Wagner’s previous results [9], our analysis is capable of capturing a discreet effect in the diffuse subtype, as well as a result for the intestinal subtype in line with the conclusions of the TAX325 RCT [7]. However, the hypothesis about the specific effect of DPF on diffuse tumors would have to be elucidated in further RCTs, which we believe to be unlikely at this time. With respect to the validity of our results, the reader must be aware that the registry still contains a limited number of subjects treated with FLOT, which is also a specific limitation in Wagner’s meta-analysis, used as a prior in our Bayesian model [9]. Despite the fact that the impact of FLOT in seniors is as yet uncertain, the Bayesian model adjusted here contemplates the type of platin, age, and year of treatment, among other factors. While more experience with this scheme in advanced tumors is still needed, the data currently available in perioperative disease point to FLOT being less active in tumors with diffuse histology, which is consistent with our results [11].

In short, our data confirm the need to update the applicability of RCTs, such as TAX325 [7], from time to time, or others that will fall behind as clinical practice changes [44], especially in view of the biological diversity of this disease. Ultimately, our study confirms the benefit of the docetaxel triple-drug regimen as first-line treatment in the real-world setting, attesting to the greater applicability in middle-aged individuals with non-diffuse tumors. However, in the best-case scenario, the benefit is modest and comes at the expense of increased toxicity. Current clinical practice guidelines endorse the use of dual-agent schedules, whereas triplets with docetaxel are deemed a useful alternative for fit patients in the event that an urgent tumor response is needed or in locally advanced, unresectable tumors [14, 45, 46]. The need to design new RCTs in AGC separating the evaluation of effects based on clinical–pathological variables is crucial given these data. This information should be taken into account when choosing treatment strategy in the context of the growing recommendation of dual-agent schedules in first line and the continuum of care of AGC.

## Electronic supplementary material

Below is the link to the electronic supplementary material.

Supplementary material 1 (PDF 27 kb)

Supplementary material 2 (PDF 14 kb)

Supplementary material 3 (PPTX 358 kb)

Supplementary material 4 (PDF 29 kb)

Supplementary material 5 (PDF 22 kb)

Supplementary material 6 (DOCX 27 kb)

Supplementary material 7 (DOCX 30 kb)

Supplementary material 8 (DOCX 15 kb)

## Data Availability

All the data generated or analyzed in this study are included in the manuscript or the supplementary information.
