# Supplementary material for: External validity of docetaxel triplet trials in advanced gastric cancer: are there patients who still benefit?
Source: Gastric Cancer. 2020 Sep 24;24(2):445–56. doi: 10.1007/s10120-020-01116-x (PMC7902567; doi:10.1007/s10120-020-01116-x)
Supplement: Supplementary file 1 — Supplementary material 1 (PDF 27 kb) [file 10120_2020_1116_MOESM1_ESM.pdf]

Annex Figure 1. Frequentist AFT model for overall survival

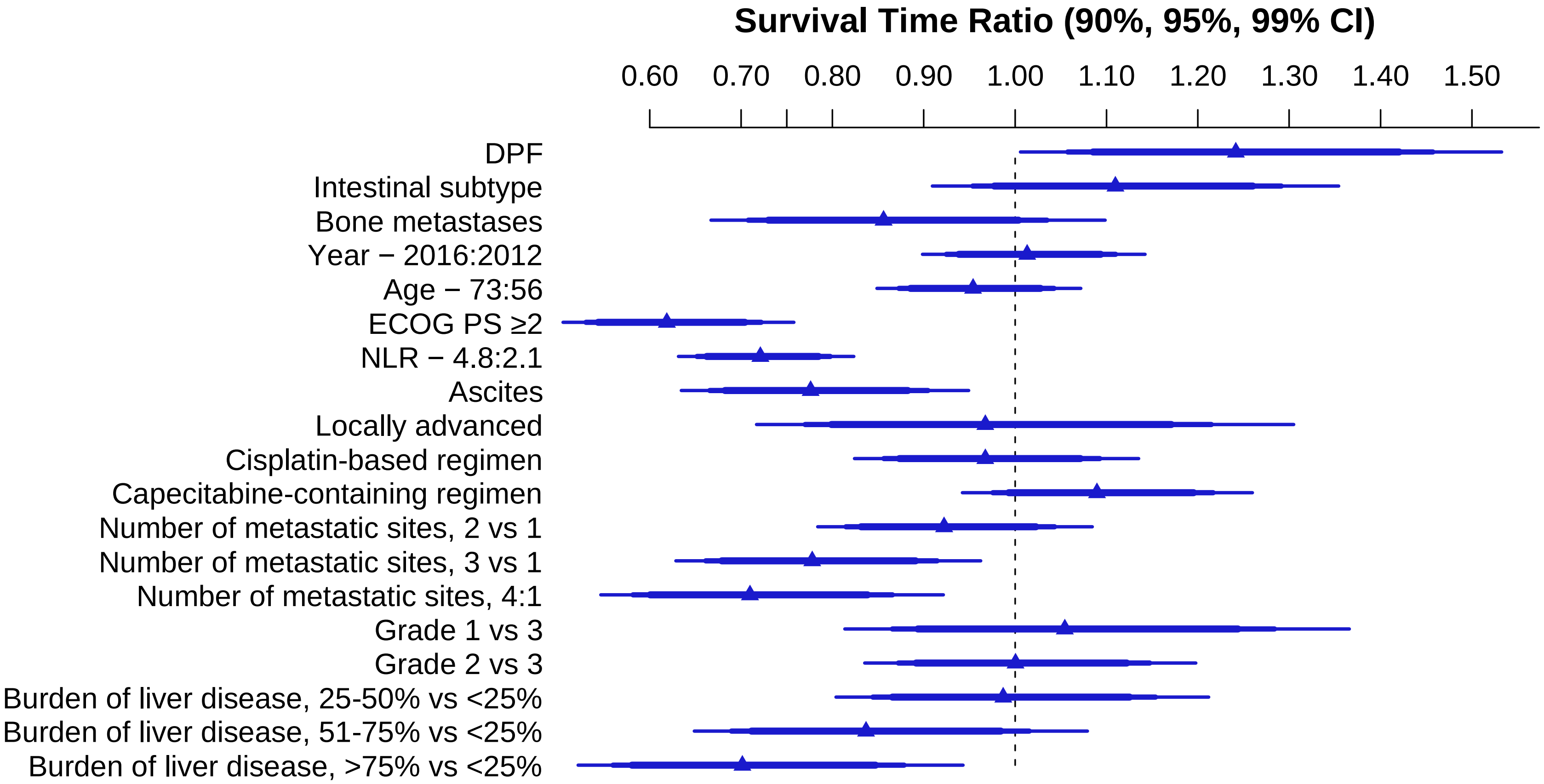

Abbreviations: AFT, accelerated failure time; DPF, docetaxel, platinum, fluoropyrimidine; ECOG-PS, Eastern Cooperative Group Performance Status; NLR, neutrophil-to-lymphocyte, CI, confident interval
