# Supplementary figures and images for: External validity of docetaxel triplet trials in advanced gastric cancer: are there patients who still benefit?
Source: Gastric Cancer. 2020 Sep 24;24(2):445–56. doi: 10.1007/s10120-020-01116-x (PMC7902567; doi:10.1007/s10120-020-01116-x)

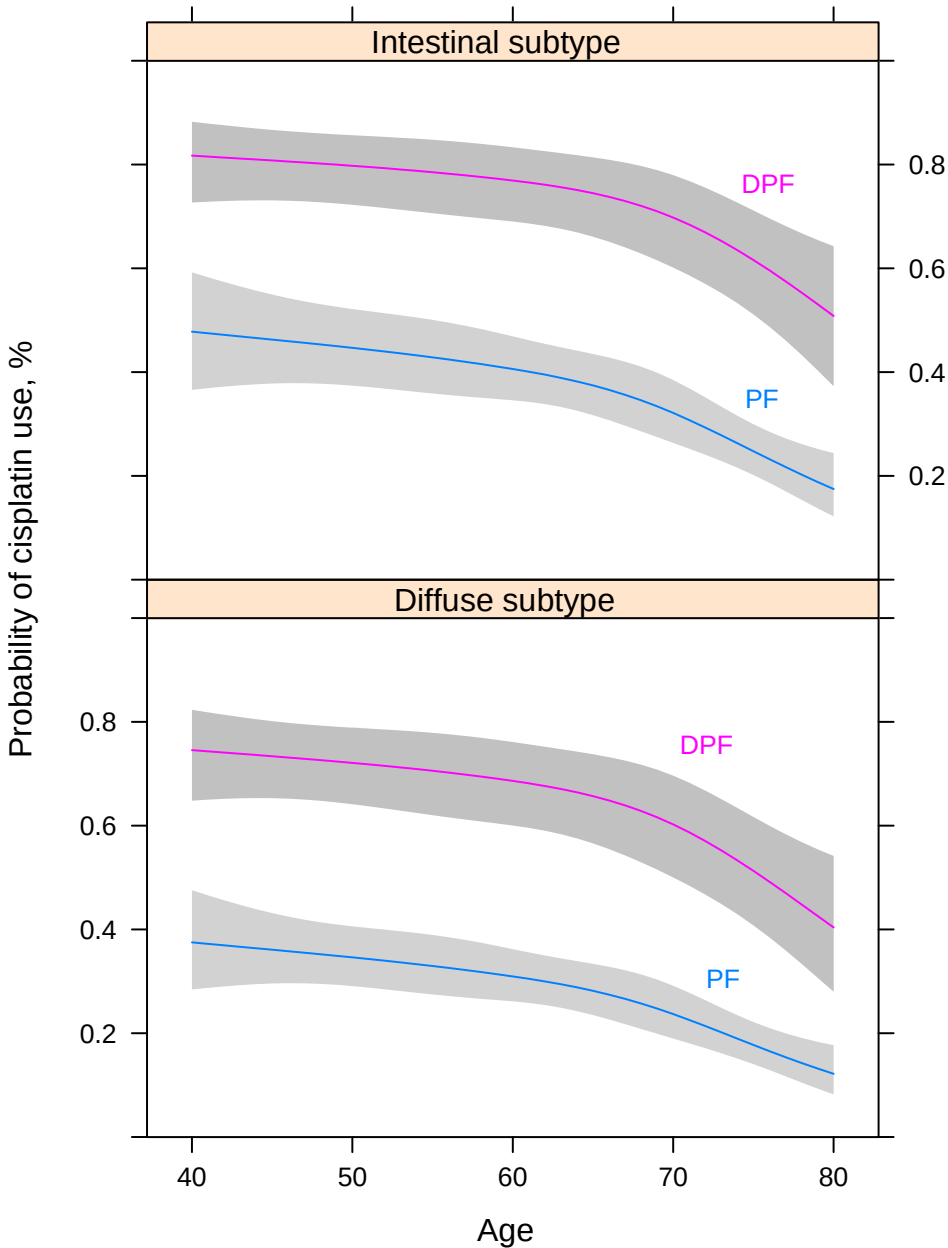

Supplement: Supplementary file 2 — Supplementary material 2 (PDF 14 kb) [file 10120_2020_1116_MOESM2_ESM.pdf]
