# Supplementary material for: External validity of docetaxel triplet trials in advanced gastric cancer: are there patients who still benefit?
Source: Gastric Cancer. 2020 Sep 24;24(2):445–56. doi: 10.1007/s10120-020-01116-x (PMC7902567; doi:10.1007/s10120-020-01116-x)
Supplement: Supplementary file 3 — Supplementary material 3 (PPTX 358 kb) [file 10120_2020_1116_MOESM3_ESM.pptx]

## Slide 1
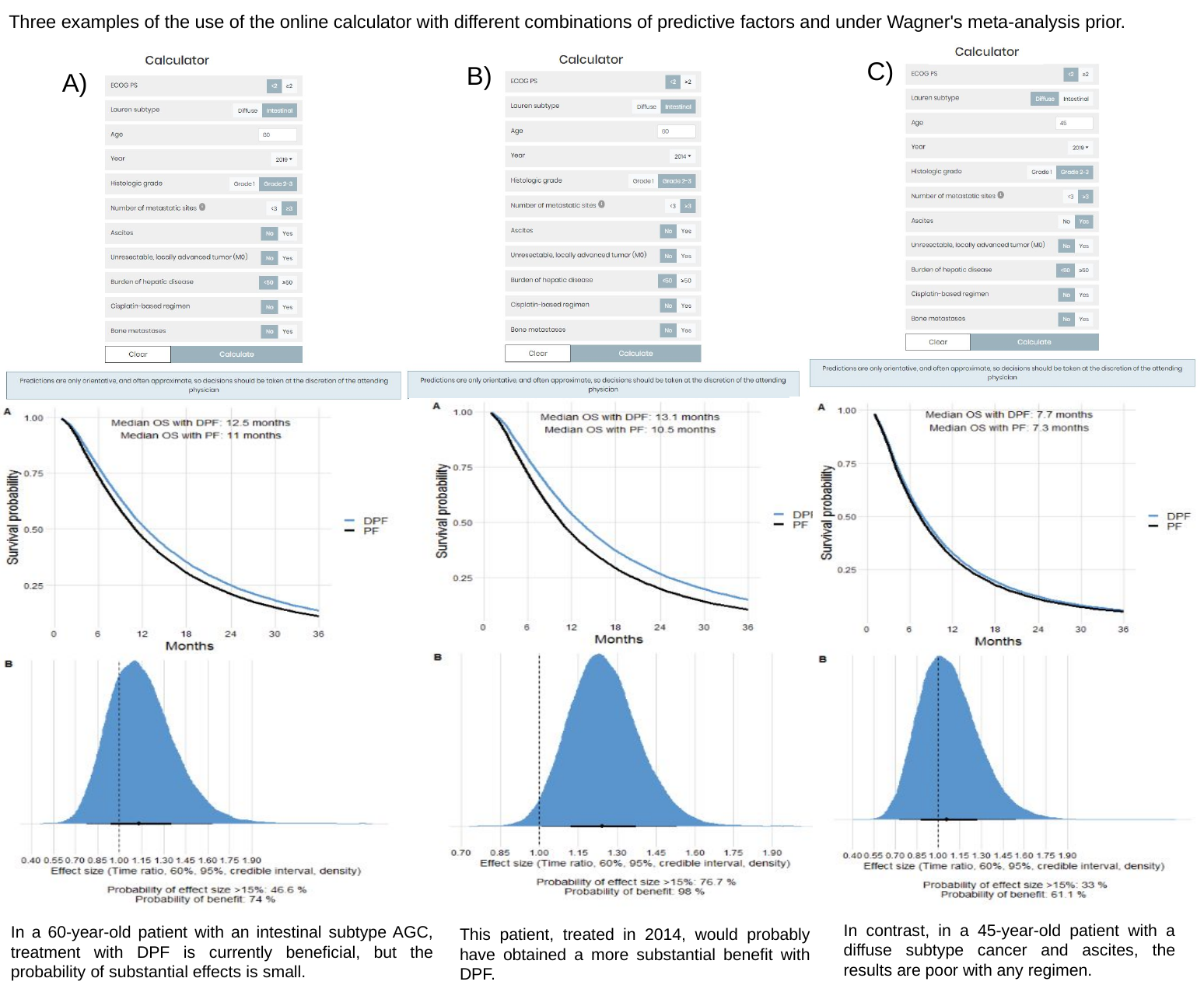

Three examples of the use of the online calculator with different combinations of predictive factors and under Wagner's meta-analysis prior.
C)
B)
A)
In contrast, in a 45-year-old patient with a diffuse subtype cancer and ascites, the results are poor with any regimen.
In a 60-year-old patient with an intestinal subtype AGC, treatment with DPF is currently beneficial, but the probability of substantial effects is small.
This patient, treated in 2014, would probably have obtained a more substantial benefit with DPF.
