# Supplementary material for: External validity of docetaxel triplet trials in advanced gastric cancer: are there patients who still benefit?
Source: Gastric Cancer. 2020 Sep 24;24(2):445–56. doi: 10.1007/s10120-020-01116-x (PMC7902567; doi:10.1007/s10120-020-01116-x)
Supplement: Supplementary file 4 — Supplementary material 4 (PDF 29 kb) [file 10120_2020_1116_MOESM4_ESM.pdf]

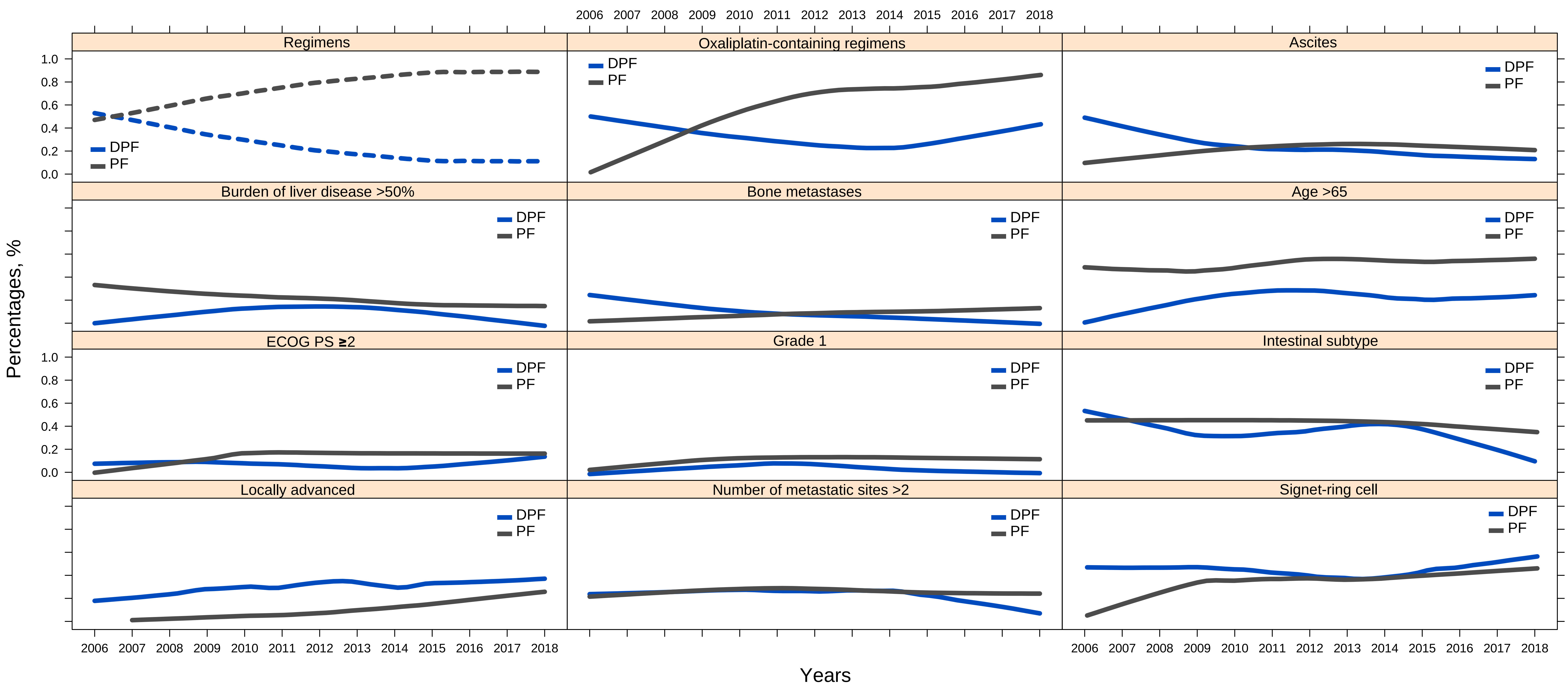

Regimens
Oxaliplatin-containing regimens
Ascites
Burden of liver disease >50%
Bone metastases
Age >65
ECOG PS ≥2
Grade 1
Intestinal subtype
Locally advanced
Number of metastatic sites >2
Signet-ring cell
DPF
PF
Percentages, %
Years
2006 2007 2008 2009 2010 2011 2012 2013 2014 2015 2016 2017 2018
0.0 0.2 0.4 0.6 0.8 1.0
