# Supplementary material for: External validity of docetaxel triplet trials in advanced gastric cancer: are there patients who still benefit?
Source: Gastric Cancer. 2020 Sep 24;24(2):445–56. doi: 10.1007/s10120-020-01116-x (PMC7902567; doi:10.1007/s10120-020-01116-x)
Supplement: Supplementary file 5 — Supplementary material 5 (PDF 22 kb) [file 10120_2020_1116_MOESM5_ESM.pdf]

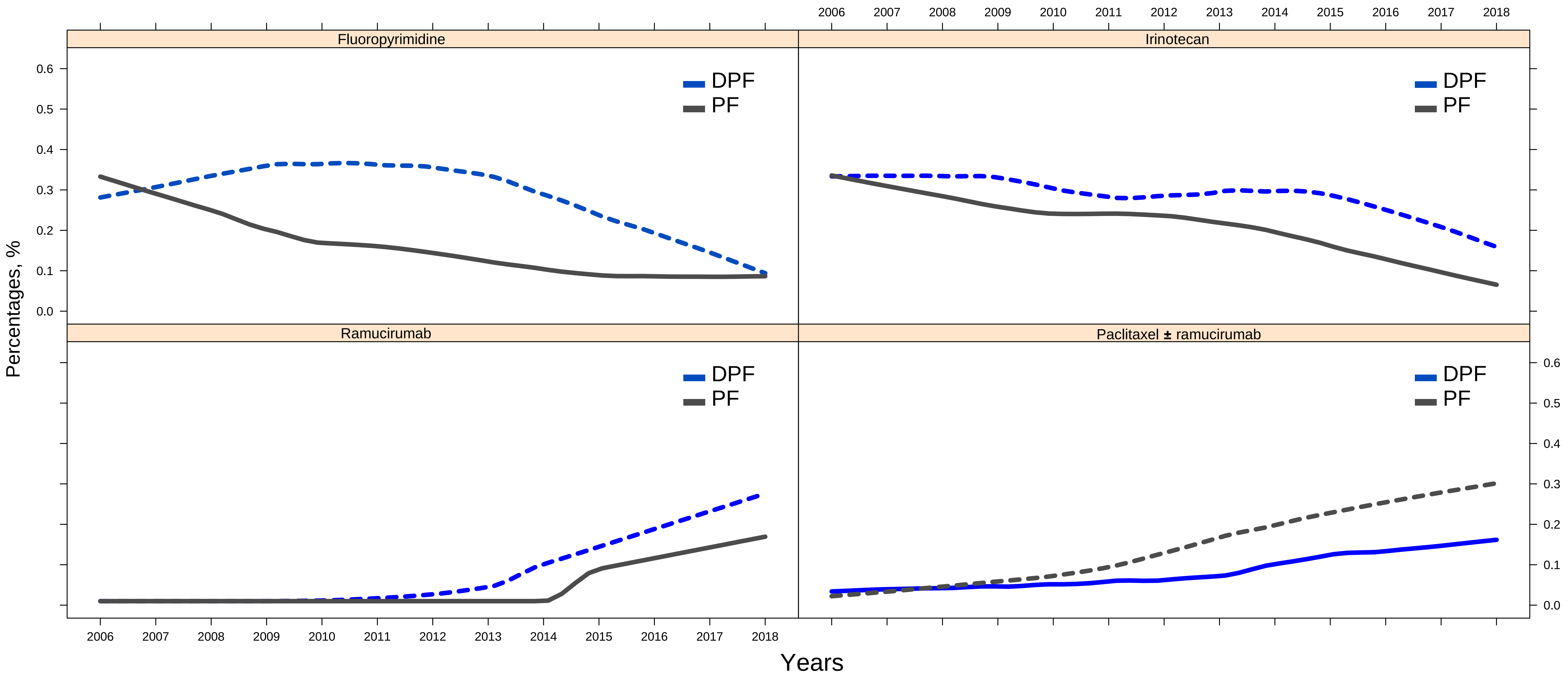

Fluoropyrimidine
Irinotecan
Ramucirumab
Paclitaxel ± ramucirumab
DPF
PF
Percentages, %
Years
0.0
0.1
0.2
0.3
0.4
0.5
0.6
2006
2007
2008
2009
2010
2011
2012
2013
2014
2015
2016
2017
2018
