# Supplementary material for: External validity of docetaxel triplet trials in advanced gastric cancer: are there patients who still benefit?
Source: Gastric Cancer. 2020 Sep 24;24(2):445–56. doi: 10.1007/s10120-020-01116-x (PMC7902567; doi:10.1007/s10120-020-01116-x)
Supplement: Supplementary file 6 — Supplementary material 6 (DOCX 27 kb) [file 10120_2020_1116_MOESM6_ESM.docx]

**Annexed Table 1** First-line chemotherapy regimens

| **Regimens with two drugs, PF doublets** | **PF doublets (n=1138)** |
| --- | --- |
| 1. **FOLFOX6**: Oxaliplatin 85 mg/m2 on day 1 + Leucovorin 400 mg/m2 on day 1 + Fluorouracil 400 mg/m2 bolus on day 1 + FU 2400 mg/m2 CI over 46 hours every 2 weeks | 355 (31%) |
| 1. **CAPOX**: Oxaliplatin 130 mg/m2 on day 1 + Capecitabine 1000 mg/m2/12h on days 1-14 every 3 weeks | 342 (30%) |
| 1. **XP**: Cisplatin 85 mg/m2 on day 1 + Capecitabine 1000 mg/m2/12h on days 1-14 every 3 weeks | 216 (19%) |
| 1. **FP3w**: Cisplatin 75 mg/m2 on day 1 + FU 750 mg/m2 CI over 24 hours daily on days 1-5 every 3 weeks | 82 (7%) |
| 1. **CAPOX biweekly modified**: Oxaliplatin 85 mg/m2 on day 1 + Capecitabine 625 mg/m2/12h daily every 2 weeks | 55 (5%) |
| 1. **Modified FUOX**: Oxaliplatin 85 mg/m2 + FU 3000 mg/m2 CI over 48 hours every 2 weeks | 41 (4%) |
| 1. **Other: Oxaliplatin-based doublets** | 13 (1%) |
| 1. **FP4w**: Cisplatin 1000 mg/m2 on day 1 + FU 1000 mg/m2 CI over 24 hours daily on days 1-5 every 4 weeks | 12 (1%) |
| 1. **Other: Cisplatin, Fluorouracil** | 8 (1%) |
| 1. **FLO**: Oxaliplatin 85 mg/m2 on day 1 + Leucovorin 200 mg/m2 on day 1 + FU 2600 mg/m2 CI over 46 hours every 2 weeks | 7 (1%) |
| 1. **Other: Oxaliplatin, Tegafur** | 6 |
| 1. **Other: Cisplatin, Tegafur** | 1 |
| **Regimens with three drugs, DPF triplets** | **DPF triplets (n=238)** |
| 1. **DCF3w**: Docetaxel 60 mg/m2 on day 1 + Cisplatin 60 mg/m2 on day 1 + FU 750 mg/m2 CI over 24 hours daily on days 1-4 every 3 weeks | 89 (38%) |
| 1. **DCX** (docetaxel, cisplatin, capecitabine): Docetaxel 75 mg/m2 on day 1 + Cisplatin 75 mg/m2 on day 1 + Capecitabine 750 mg/m2/12h on days 1-14 every 3 weeks | 59 (25%) |
| 1. **FLOT**: Oxaliplatin 85 mg/m2 on day 1 + leucovorin 200 mg/m2 on day 1 + FU 2600 mg/m2 CI over 24 hours + Docetaxel 50 mg/m2 on day 1 every 2 weeks | 40 (17%) |
| 1. **DOX** (docetaxel, oxaliplatin, capecitabine): Docetaxel 75 mg/m2 on day 1 + Oxaliplatin 100 mg/m2 on day 1 + Capecitabine 750 mg/m2/12h on days 1-14 every 3 weeks | 35 (15%) |
| 1. **DCF4w**: Docetaxel 75 mg/m2 on day 1 + Cisplatin 75 mg/m2 on day 1 + FU 1000 mg/m2 CI over 24 hours daily on days 1-5 every 4 weeks | 15 (6%) |

Abbreviations: CI, continuous infusion; FU, fluorouracil; w, week.
