# Supplementary material for: External validity of docetaxel triplet trials in advanced gastric cancer: are there patients who still benefit?
Source: Gastric Cancer. 2020 Sep 24;24(2):445–56. doi: 10.1007/s10120-020-01116-x (PMC7902567; doi:10.1007/s10120-020-01116-x)
Supplement: Supplementary file 7 — Supplementary material 7 (DOCX 30 kb) [file 10120_2020_1116_MOESM7_ESM.docx]

**Annex Table 2** Doses of oxaliplatin, cisplatin, fluorouracil and docetaxel in frequent regimens

| **Drug** | **Oxaliplatin** | | | **Cisplatin** | | | **FU CI** | | **Docetaxel** |
| --- | --- | --- | --- | --- | --- | --- | --- | --- | --- |
| **Regimen** | **FLOT** | **FOLFOX6** | **CAPOX** | **XP** | **FP3w** | **DCX/ DCF*** | **FOLFOX6** | **DCF/FLOT** | **DCX/DCF/**  **DOX/FLOT*** |
| **Number of cycles**  **(median, range)** | 6 (2-12) | 8 (1-59) | 5 (1-19) | 6 (1-29) | 5 (1-6) | 6 (1-19) | 9 (1-60) | 6 (1-27) | 6 (1-19) |
| **Median of treatment duration (weeks)** | 17 | 20 | 18 | 18 | 18 | 18 | 22 | 18 | 18 |
| **Mean cumulative dose (mg/m2)** | 584 | 661 | 644 | 393 | 362 | 323 | 22616 | 18396 | 325 |
| **Mean dose/cycle (mg/m2/cycle)** | 84 | 80 | 120 | 74 | 71 | 62 | 2287 | 2929 | 61 |
| **Mean dose intensity**  **(mg/m2/week)** | 34 | 35 | 37 | 22 | 21 | 18 | 997 | 957 | 19 |
| **Relative dose intensity** | 80% | 82% | 86% | 81% | 77% | 78% | 83% | 74% | 82% |
| **Reason of withdrawal**  ***Toxicity***  ***Progression***  ***Planned treatment completed***  ***Patient refusal***  ***Other***  ***Not available*** | 6%  32%  35%  3%  10%  13% | 22%  40%  17%  3%  11%  6% | 25%  41%  21%  2%  8%  4% | 16%  52%  29%  1%  2%  0 | 9%  48%  33%  1%  5%  5% | 15%  40%  35%  3%  6%  1% | 7%  50%  19%  5%  11%  8% | 5%  53%  28%  2%  8%  3% | 14%  39%  36%  3%  7%  2% |

Abbreviations: CAPOX, capecitabine, oxaliplatin; DCF, docetaxel, cisplatin, fluorouracil; DCX, docetaxel, cisplatin, capecitabine; DOX, docetaxel, oxaliplatin, capecitabine; FLOT, fluorouracil, oxaliplatin, docetaxel; FOLFOX6, fluorouracil, oxaliplatin; FP3w, fluorouracil, cisplatin every 3 weeks XP, capecitabine, cisplatin.

* Reference dose used for cisplatin/docetaxel: 70 mg/m2/3-weeks.
