# Supplementary material for: External validity of docetaxel triplet trials in advanced gastric cancer: are there patients who still benefit?
Source: Gastric Cancer. 2020 Sep 24;24(2):445–56. doi: 10.1007/s10120-020-01116-x (PMC7902567; doi:10.1007/s10120-020-01116-x)
Supplement: Supplementary file 8 — Supplementary material 8 (DOCX 15 kb) [file 10120_2020_1116_MOESM8_ESM.docx]

**Annex Table 3** Evaluation of potentially age-dependent effect-modifying factors (under weakly informative priors)

| **Variables** | **Time ratio**  **(95%, CrI)** | **Posterior probability of effect size >15%**  **(TR >1.15)** | **Posterior probability of effect size >30%**  **(TR >1.30)** | **Probability of interaction outside ROPE** |
| --- | --- | --- | --- | --- |
| **Organs involved, 4** | 1.22 (1.04-1.43) | 73% | 26% | 24% |
| **Organs involved, 3** | 1.18 (1.02-1.39) | 65% | 17% | 17% |
| **Organs involved, 2** | 1.27 (1.10-1.44) | 88% | 37% | 37% |
| **Organs involved, 1** | 1.17 (1.09-1.24) | 67% | 0 | - |
| **Intestinal subtype** | 1.27 (1.08-1.11) | 90% | 40% | 41% |
| **Diffuse subtype** | 1.17 (1.09-1.24) | 67% | 0 | - |
| **Grade 3** | 1.19 (1.06-1.36) | 72% | 14% | 10% |
| **Grade 2** | 1.20 (1.05-1.40) | 73% | 22% | 18% |
| **Grade 1** | 1.17 (1.09-1.25) | 69% | 1% | - |
| **ECOG PS ≥2** | 1.16 (0.99-1.36) | 54% | 1% | 20% |
| **ECOG PS 0-1** | 1.18 (1.10-1.25) | 75% | 15% | - |
| **Burden of liver disease >75%** | 1.16 (0.98-1.37) | 53% | 13% | 21% |
| **Burden of liver disease 51-75%** | 1.19 (1.02-1.40) | 66% | 20% | 20% |
| **Burden of liver disease 25-50%** | 1.16 (0.99-1.36) | 54% | 12% | 19% |
| **Burden of liver disease <25%** | 1.18 (1.10-1.25) | 74% | 1% | - |

Abbreviations: CrI, credible interval; ECOG-PS, Eastern Cooperative Group Performance Status: TR, adjusted time ratios.

Note: A time ratio of more than 1 for the covariate implies that this slows or prolongs the time to event, whereas a time ratio of less than 1 indicates that an event is more likely to occur earlier. Thus, a time ratio equal to 2 would mean that the median of time to event is doubled in patients treated with DPF.

The posterior probability of effect sizes >15% or 30% (TR >1.15 or 1.30) denotes the actual probability of achieving a benefit of that magnitude (15 or 30%) or greater.
